# Expression analysis of several antiviral related genes to BmNPV in different resistant strains of silkworm, *Bombyx mori*

**DOI:** 10.1093/jis/14.1.76

**Published:** 2014-01-01

**Authors:** Yang Cheng, Xue-yang Wang, Chang Du, Juan Gao, Jia-ping Xu

**Affiliations:** School of Life Sciences, Anhui Agricultural University, Hefei 230036, China

**Keywords:** *Bombyx mori* nucleopolyhedrovirus, *Bmlipase-1*, *BmNox*, *Bmserine protease-2*, expression analysis

## Abstract

*Bombyx mori* L. (Lepidoptera: Bombycidae) nucleopolyhedrovirus (BmNPV) is a highly pathogenic virus in the sericultural industry, often causing severe damage leading to large economic losses. The immune mechanisms of *B. mori* against this virus remain obscure. Previous studies had demonstrated Bmlipase-1, BmNox and Bmserine protease-2 showing antiviral activity *in vitro* , but data on the transcription levels of these proteins in different resistant strains were not reported. In order to determine the resistance level of the four different strains (P50, A35, A40, A53) and gain a better understanding of the mechanism of resistance to BmNPV in *B. mori* , the relative expression level of the genes coding the three antiviral proteins in larval haemolymph and midgut of different *B. mori* strains resistant to BmNPV was determined. The results showed that these genes expressed significantly higher in the resistant strains compared to the susceptible strain, and the differential expression levels were consistent with the LC50 values in different strains. The transcription level of the target genes almost all up-regulated in the larvae midgut and down-regulated in the haemolymph. The results indicate the correlation of these genes to BmNPV resistance in *B. mori.*

## Introduction

The silkworm, *Bombyx mori* L. (Lepidoptera: Bombycidae), is a commercially important insect for production of silk and recombinant proteins and is also a good model of the Lepidoptera ( Goldsmith et al. 2005 ). *Bombyx mori* nucleopolyhedrovirus (BmNPV) is a primary pathogen of the domestic silkworm and causes large economic losses ( Miao et al. 2005 ). Most silkworm strains are highly susceptible to BmNPV, while only a few exhibit high resistance to BmNPV ( Bao et al. 2009 ). The heredity of silkworm resistance to BmNPV is relatively complicated because it is controlled both by major dominant genes and multiple micro-effective genes ( Yao et al. 2003 ). In insects, the midgut is involved in defense responses against pathogens, meanwhile it has been shown that BmNPV could invade the fat body and haemocytes of both susceptible and resistant strains through the injection route, but viral proliferation in the fat body and haemocytes of the resistant strain was notably slowed ( Bao et al. 2010 ).

A number of studies have been conducted on insect resistance to NPV. *Bombyx mori* serine protease-2, lipase-1, and alkaline trypsin protein purified from the digestive juice of *B. mori* larvae showed strong antiviral activity to BmNPV *in vitro* ( Ponnuvel et al. 2003 ; Nakazawa et al. 2004; Ponnuvel et al. 2012 ). Soluble *B. mori* NADPH oxidoreductase (BmNox) isolated from silkworm larvae gut juice was also confirmed to have antiviral activity to BmNPV ( Selot et al. 2007 ; Selot et al. 2010 ). Recently, comparative gene expression techniques, such as differential display, cDNA microarray assay, and two-dimensional gel electrophoresis (2-DE), have become routine to examine changes in gene expression. Using the fluorescent differential display (FDD) technique, *Bmsop2* and *Bms3a* were identified to be related to BmNPV resistance in silkworm ( Xu et al. 2005 ; Xu et al. 2008 ). A gene encoding arginine kinase in *B. mori* has been identified differentially expressed in the midgut of *B. mori* resistant and susceptible strains to BmNPV by 2-DE ( Kang et al. 2011 ). But, the transcription level of the mentioned genes in different resistant strains was not confirmed accurately.

In this study, the expression of several previously reported antiviral genes in the haemolymph and midgut of silkworm strains differing in virus resistance was quantified using realtime quantitative PCR to confirm their relevance to antiviral activity.

## Materials and Methods

### Silkworm strains and virus

Long-established silkworm strains, including a low resistant strain P50 (a standard reference strain), a highly resistant strain A35, and strains A40 and A53, which were previously uncharacterized for viral resistance, were maintained in the Key Laboratory of Sericulture, Anhui Agricultural University, Hefei, China. The first three instars of larvae (P50, A35, A40, and A53) were reared on fresh mulberry leaves at 26 ± 1°C, 75 ± 5% of relative humidity, with a 12:12 L:D photoperiod, and the last two instars were reared on fresh mulberry leaves at 24 ± 1°C under the same relative humidity and photoperiod as before.

BmNPV T3 strain ( Gomi et al. 1999 ) was maintained in our laboratory. The occlusion body (OB) of T3 strain was obtained from the haemolymph of infected larvae and was purified by repeated and differential centrifugation following the previously published protocol ( Rahman et al. 2004 ). The concentration of the virus (OB/mL) was determined using a haemocytometer (Hujiang Medical Instruments, ( www.shhjylqx.cn ). The suspension in sterile distilled water was prepared to contain 1×108 OB/mL. The desired concentrations of OB suspension were prepared by serial dilution in sterile distilled water.

### Silkworm strain resistance level bioassays

The fourth instar *B. mori* larvae were used in oral infection assays to determine the infectivity of BmNPV. Fresh mulberry leaves were cut into 1 cm × 1 cm pieces, and 30 µL of BmNPV T3 strain with different concentrations (1×108 OB/mL, 1×107 OB/mL, 1×106 OB/mL, 1×105 OB/mL, and 1×104 OB/mL) was spread on each piece. Every larva was fed with a piece of aired mulberry leaf, then was transferred to fresh mulberry leaves and reared at 24 ± 1°C after the small piece was eaten, and the mortality was investigated every 24 hours. Every 30 larvae were used in each of the three independent experiments. The infectivity of BmNPV was determined by probit analysis with IBM SPSS Statistics 20 (IBM, ( www.ibm.com ).

### Experimental treatments of silkworm

Thirty fifth instar molt larvae chosen from each strain were divided into three groups and starved overnight. All the 120 larvae were infected with 500 OB of BmNPV T3 strain per larva orally. The 120 control larvae were fed with 5 µL sterile distilled water instead of OB. The infected and control groups were reared in different rooms under the same condition.

### Preparation of haemolymph and midgut tissues of silkworm larvae

Based on the reported time course of viral proliferation and expression of NPV responsive genes in the fat body and haemocytes of susceptible and resistant strains ( Bao et al. 2010 ), at 48 hr the caudal horns of the larvae were cut and the haemolymph was collected using microcentrifuge tubes (Kirgen, ( www.kirgen.com ) that contained a small aliquot of phenylthiocarbamide to protect the haemolymph from oxidation. 500 µL of haemolymph was mixed with an equal volume of TRIzol Reagent (Invitrogen, Life Technologies, ( www.lifetechnologies.com ) then homogenized using a pellet pestle motor (Kontes, ( www.kontes.cz ) and stored at −80°C for later use. Midguts were taken from the horn cut larvae and dissected in PBS (137 mM NaCl, 2.7 mM KCl, 4.3 mM Na2HPO4, and 1.4 mM KH2PO4) prepared with DEPC-reated H2O (Sangon, ( www.sangon.com ). Each tube contained 100 mg wet weight midgut, was mixed with 1000 µL TRIzol reagent, and was then homogenized using a pellet pestle motor and stored at −80°C for later use.

### RNA isolation and cDNA synthesis

Total RNA was extracted from *B. mori* haemolymph and midgut using TRIzol reagent according to the manufacturer’s instructions. The ratios of A260/280 and the concentrations for the RNA samples were determined using a NanoDrop 2000 spectrophotometer (Thermo Fisher Scientific, ( www.thermofisher.com ).

Total RNA samples were treated with RT reagent kit with gDNA Eraser (TaKaRa, ( www.takara.com.cn ) to remove genomic DNA and synthesize the first strand cDNA according to the manufacturer’s instructions. Briefly, 2.0 µL 5×g DNA Eraser buffer, 1.0 L gDNA Eraser, and 1.0 µg total RNA were ixed in a 200 µL PCR tube and then RNase Free dH2O was added up to 10 µL, and the solution was then incubated at room temperature for 5 minutes. 4.0 µL 5× PrimeScript buffer, 1.0 µL PrimeScript RT Enzyme Mix I, and 1.0 µL RT Primer Mix was then added to the previous tube, then RNase Free dH2O was added up to 20 µL, and the solution was then incubated at 37°C for 15 minutes followed by 85°C for 5 seconds and stored at −20°C for later use.

### PCR primer design

Three target genes, *Bmlipase-1* (GenBank accession no. AB076385.1), *Bmserine protease-2* (GenBank accession no. AB073673.1), and *BmNox* (GenBank accession no. EF025315.2), related to antiviral activity against BmNPV were analyzed using the following primers:

5'-CTGGAACAGCAACGGAAACT-3' (forward primer) and 5'-TCCGTTGTTGATGAGCCAGA-3' (reverse primer);

5'-CCAGGATTGTGGGTGGTTCT-3' (forward primer) and 5'-CAAAAGCGAGGGTGAACTGA-3' (reverse primer);

5'-CGACGAGCGACTAACCCAAA-3' (forward primer) and 5'-GAGCTCATACGGACTCAACCTG-3' (reverse primer).

As an internal control, the *Bombyx mori ribosomal protein s3* ( *Bmrps3* ) gene (GenBank accession no. AY769316.1) was also analyzed using the forward primer:

5'-CGATTCAACATTCCAGAGCA-3' and the reverse primer: 5'-GAACACCATAGCAAGCACGAC-3'. Primers were designed using Primer Premier 5.0 (Premier Biosoft, ( www.premierbiosoft.com ).

### Expression analysis of antiviral genes by realtime PCR

Realtime quantitative PCR was carried out in a 25 µL reaction mix containing 12.5 µL of SYBR Premix Ex Taq (TaKaRa, ( www.takara.com.cn ), 1 µL of 1:5 diluted cDNA template, 1 µL of each of the primers (10 µM), and 9.5 µL ddH2O. The thermal cycling profile consisted of initial denaturation at 95°C for 30 sec and 40 cycles at 95°C for 5 sec, 60°C for 30 sec, and 72°C for 20 sec. Relative expression levels were calculated using the 2−∆∆Ct method, where ∆∆Ct=∆Ctsample−∆Ctreference following the previously published protocol ( Livak and Schmittgen 2001 ). PCR reactions were performed in 96-well plates with a Multicolor Realtime PCR Detection System (Bio-Rad, ( www.bio-rad.com ) using SYBR Green to detect dsDNA synthesis. PCR amplification was performed in duplicate wells.

### Statistical analysis

The infectivity of BmNPV was determined by probit analysis with IBM SPSS Statistics 20 (larval mortality was set as the response frequency, the larvae number as the total observed, the BmNPV concentrations as the covariate(s), and the transform option was set as Log base 10). The relative expression level of the three target genes were analyzed by multiple ANOVA with SAS 9.3 (SAS Institute, ( www.sas.com ).

## Results

### BmNPV infectivity in different silkworm strains

The LC50 value of A35 was about 50 fold greater than that of A40 and A53, and over 500 fold greater than that of P50. The value of A40 was a little higher than that of A53, but the difference was not significant ( Table 1 ).

**Table 1.**
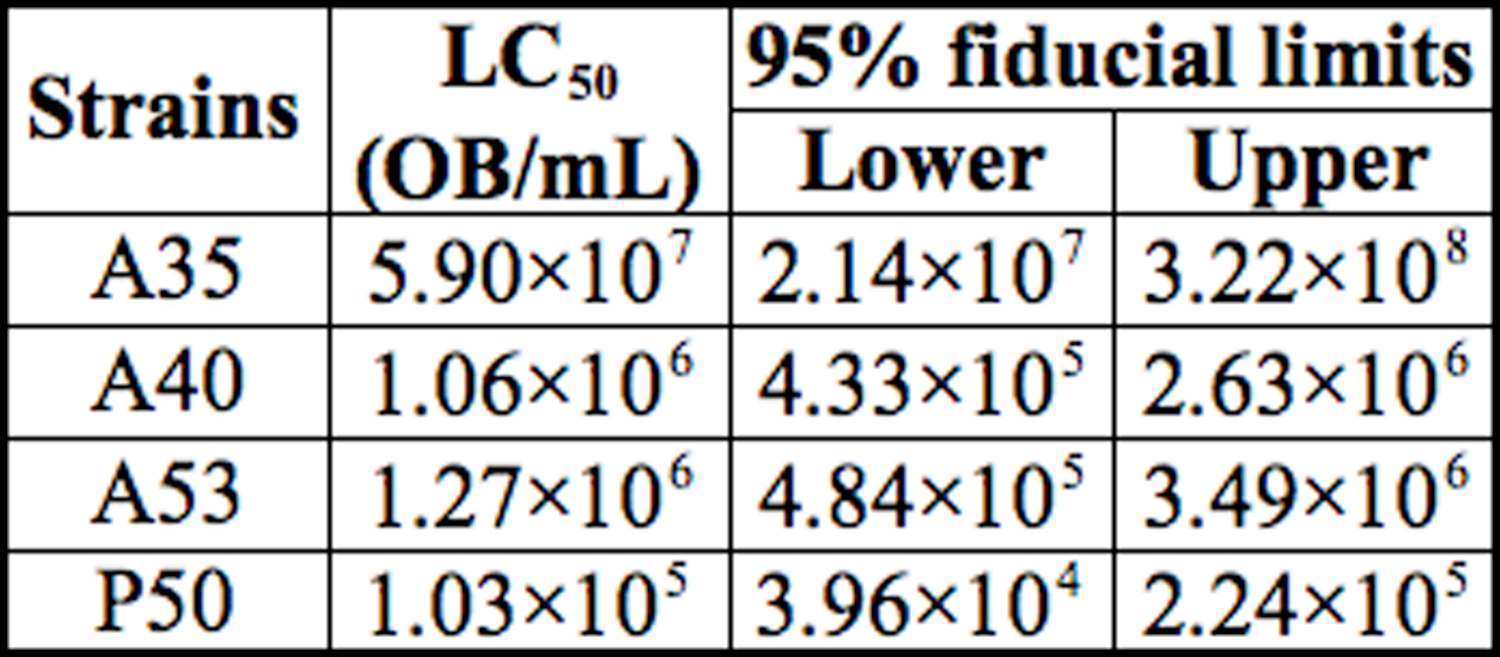
Concentration mortality of *Bombyx mori* larvae infected with BmNPV.

### Realtime quantitative PCR analysis

**Figure 1.**
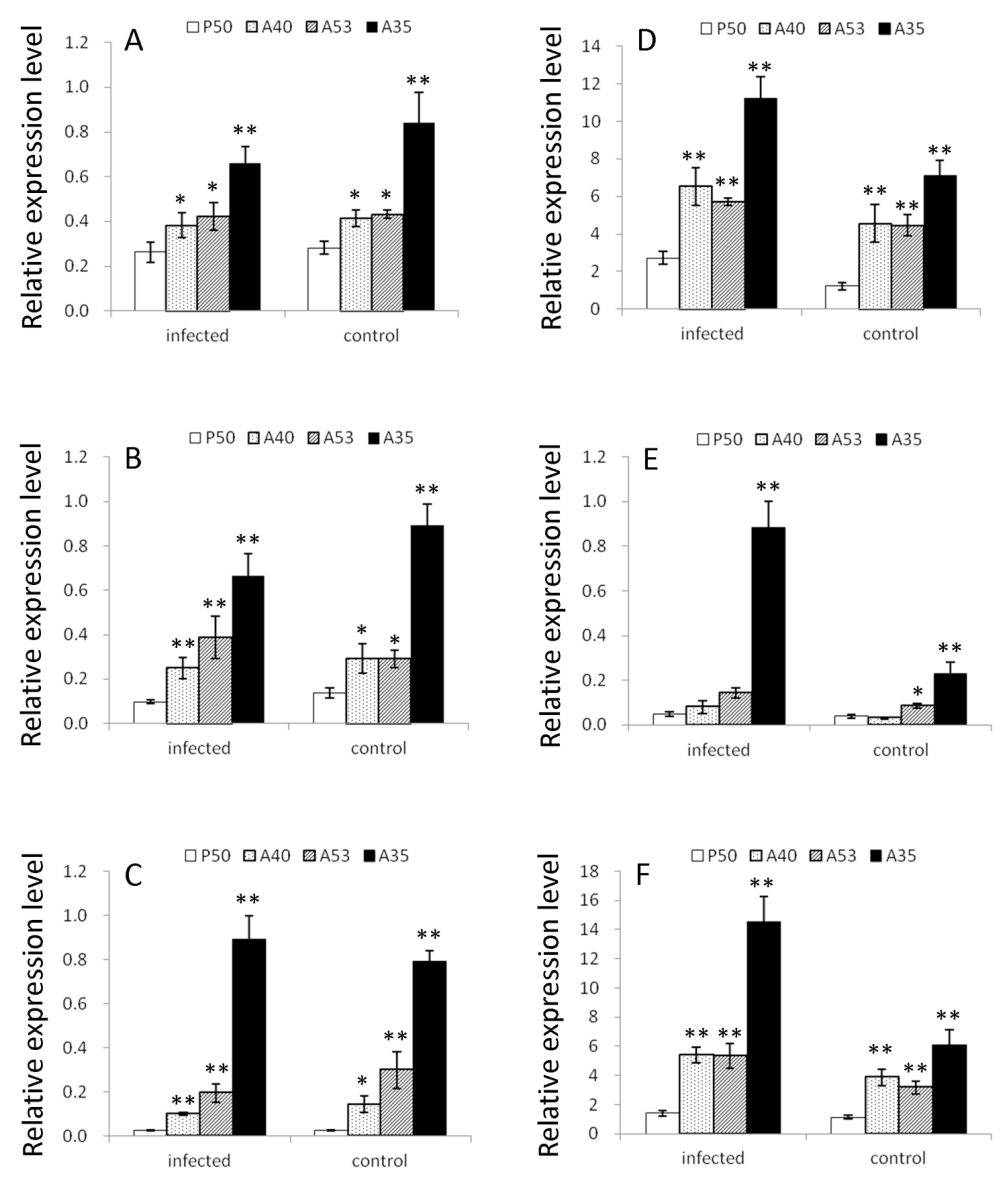
Relative expression level of antiviral genes in *Bombyx mori* hemolymph and midgut of different strains. A, B, and C refer to relative expression levels of *Bmlipase-1* , *BmNox* and *Bmserine protease-2* in hemolymph, respectively. D, E, and F refer to the same, but in the midgut. The bars from left to right indicate P50, A40, A53, and A35 respectively. *, *P* < 0.05; **, *P* < 0.01 (ANOVA and LSD aposteriori test). Error bars ± SEM. High quality figures are available online.

In order to determine the expression differences of the antiviral genes, analysis of the larval haemolymph and midgut of different *B. mori* strains was examined by realtime quantitative PCR. In the larval haemolymph, the relative expression level of *Bmlipase-1* in the A35 strain was notably up-regulated compared to the P50 strain ( *P* < 0.01) in both infected and control groups, and the A40 and A53 strains were also up-regulated compared to the P50 strain ( *P* < 0.05) ( Figure 1A ). The transcripts of *BmNox* and *Bmserine protease-2* showed a similar situation in A35, A40, and A53 strains in both infected and control groups (Figures 2A, 3A). A40 and A53 did not show significant differences in the expression of *Bmlipase-1* , *BmNox* control group, or *Bmserine protease-2* infected group, and notably decreased compared to the A35 strain ( Figure 1A–C ).

In the larval midgut, *Bmlipase-1* in A35, A40, and A53 strains all expressed differently compared to the P50 strain, and the changes were significantly high ( *P* < 0.01) in both infected and control groups ( Figure 1D ). For *BmNox* , the transcription level in A35 was significantly higher than the P50 strain ( *P* < 0.01), was not significant in the A53 infected group and A40 infected and control groups, and increased significantly in the A53 control group compared to the P50 strain ( *P* < 0.05) ( Figure 1E ). The relative expression levels of *Bm-serine protease-2* in both infected and control populations of A35, A40, and A53 strains were significantly higher than those of the P50 strain ( *P* < 0.01) ( Figure 1F ).

We also analyzed the expression differences between the infected and control groups for all four strains and found that the relative expression level of the three target genes all decreased in the larval haemolymph after BmNPV infection, except *BmNox* in A53 and *Bmserine protease-2* in A35, but the up and down regulation were not significant ( Figure 1A –C). In the larval midgut, the expression of the target genes up-regulated after BmNPV infection, and the changes were significant in A40, A53, and A35; the upregulation of *Bmserine protease-2* was significant ( *P* < 0.01) in the P50 strain, but *BmNox* and *Bmlipase-1* were not ( Figure 1D –F).

## Discussion

Based on the previous studies, we carried out gene expression analysis to three identified proteins (Bmlipase-1, BmNox, Bmserine protease-2) related to BmNPV-resistance in silkworm on the transcription level.

Bmlipase-1, a protein first purified by Ponnuvel et al. (2003) from the digestive juice of silkworm larvae, which showed 56% homology with *Drosophila melanogaster* lipase and had lipase activity *in vitro* , is representative of such protein. It was reported that overexpressing the *Bmlipase-1* gene in silkworm reduced the mortality of the transgenic line by approximately 33% compared to the nontransgenic line when the virus dose was 106 OB/larva ( Jiang et al. 2012 ). In the current study, the relative expression level of *Bmlipase-1* in resistant strains increased significantly compared to the susceptible strain in the larval haemolymph and midgut ( Figure 1A , D). As expected, the transcription level of *Bmlipase-1* between A40 and A53 did not have a significant difference, while the levels in A40 and A53 were down-regulated significantly compared to that in A35. These results corresponded to the LC50 values ( Table 1 ) and were similar to previous research ( Ponnuvel et al. 2003 ; Jiang et al. 2012 ). Du et al. (2012) identified four lipases directly involved in PBAN-stimulated sex pheromone biosynthesis in *Bombyx mori* using the DGE and RNAi approaches, but there is no evidence demonstrating the relevance between PBAN and viral resistance in insects.

*B. mori* NADPH oxidoreductase, a 26.5 kDa protein identified by Selot et al. (2007) displaying anti-BmNPV activity in BmN cell lines, was given such a name because of the homology with NADPH oxidoreductase and NADPH oxidase activity. Using western blotting and semi-quantitative expression, Selot et al. (2010) found this protein existed in the larval midgut but not in the fat body, silk gland, hemolymph, or hemocyte, and there was not any difference in the gut fluid of resistant and susceptible strains, with the exception that the anterior midgut of the susceptible strain showed a total absence of BmNox expression. In our study, *BmNox* expression level was determined much more accurately by qRT-PCR, and the existence at the transcription level of *BmNox* in larval haemolymph was confirmed, which was not in contradiction with the previous research, probably because of the mRNA transport from hemocyte to other tissues. Using qRT-PCR, it was determined that the *BmNox* relative expression level in the A35 strain was approximately 6.9 and 6.6 fold greater than that of the P50 strain in the infected and control groups of larval haemolymph respectively, and 18.1 and 7.4 fold great in larval midgut, respectively.

Bmserine protease-2 was a midgut-specific protease and could significantly reduce the infectivity of BmNPV ( Nakazawa et al. 2004 ). As reported by Qin et al. (2012) , it was expressed significantly high in BmNPV-resistant silkworms, but not in BmNPV-susceptible silkworms based on 2-DE and western blotting. Yao et al. (2008) also found that this protease only expressed in midgut, not in silk gland, fat body, hemocyte, or trachea of *Bombyx mandarina* . According to the data we obtained, the relative expression level of *Bmserine protease-2* in larval haemolymph was much lower than that in midgut (data not shown). The data also told us that the relative expression level in resistant strains was significantly higher than in the susceptible strains ( Figure 1C , F). This situation was similar to that of *Bmlipase-1* and corresponded to the LC50 values ( Table 1 ).

BmNPV infection could lead to upregulation of *Bmlipase-1* , *BmNox* , and *Bmserine protease-2* expression in the larval midgut ( Figure 1D –F) and down-regulate the expression in the haemolymph ( Figure 1A –C), suggesting midgut was the major immune barrier against BmNPV in *B. mori* . The decline of expression in the haemolymph possibly occurred because the viral infection damaged physical functions, resulting in the reduction of most of the gene expression.
